# Electrochemical Performance of Micropillar Array Electrodes in Microflows

**DOI:** 10.3390/mi11090858

**Published:** 2020-09-17

**Authors:** Bo Liu, Chuanwen Lv, Chaozhan Chen, Bin Ran, Minbo Lan, Huaying Chen, Yonggang Zhu

**Affiliations:** 1 Center for Microflows and Nanoflows, Harbin Institute of Technology (Shenzhen), Shenzhen 518000, China; 19b958013@stu.hit.edu.cn (B.L.); 19b958014@stu.hit.edu.cn (C.L.); chaozhanchen@foxmail.com (C.C.); ranbin1992@gmail.com (B.R.); chenhuaying@hit.edu.cn (H.C.); 2 Shanghai Key Laboratory of Functional Materials Chemistry, School of Chemistry and Molecular Engineering, East China University of Science and Technology, Shanghai 200237, China; minbolan@ecust.edu.cn

**Keywords:** microchip-based electrochemical detection system, micropillars array electrode, numerical simulation, tail effect

## Abstract

The microchip-based electrochemical detection system (μEDS) has attracted plenty of research attention due to its merits including the capability in high-density integration, high sensitivity, fast analysis time, and reduced reagent consumption. The miniaturized working electrode is usually regarded as the core component of the μEDS, since its characteristic directly determines the performance of the whole system. Compared with the microelectrodes with conventional shapes such as the band, ring and disk, the three-dimensional (3D) micropillar array electrode (μAE) has demonstrated significant potential in improving the current response and decreasing the limits of detection due to its much larger reaction area. In this study, the numerical simulation method was used to investigate the performance of the μEDS, and both the geometrical and hydrodynamic parameters, including the micropillars shape, height, arrangement form and the flow rate of the reactant solution, were taken into consideration. The tail effect in μAEs was also quantitatively analyzed based on a pre-defined parameter of the current density ratio. In addition, a PDMS-based 3D μAE was fabricated and integrated into the microchannel for the electrochemical detection. The experiments of cyclic voltammetry (CV) and chronoamperometry (CA) were conducted, and a good agreement was found between the experimental and simulation results. This study would be instructive for the configuration and parameters design of the μEDS, and the presented method can be adopted to analyze and optimize the performance of nanochip-based electrochemical detection system (nEDS).

## 1. Introduction

A microchip-based electrochemical detection system (μEDS), which is developed on the basis of electrochemical methods and microfluidic techniques, has demonstrated satisfactory benefits including automation, compatibility, fast analysis time, reduced reagent consumption, high sensitivity and strong specificity [1,2,3,4], and has been widely used for various on-site real-time applications [5] as well as point-of-care diagnosis [6,7,8].

Electrochemical detection (ED) is carried out based on the redox reaction of underivatized electroactive species occurring at the electrode surface [9]. Hence the miniaturized working electrode is usually regarded as a core component, of which the characteristic is directly related to the performance of the whole μEDS system. In previous researches, most of the microelectrodes were designed into a simple two-dimensional (2D) planar band [10], disk [11,12], ring [13] or slightly more complicated shapes such as hemisphere [14], cylinder [15] and ring [16]. Besides these conventional configurations, the three-dimensional (3D) microelectrodes array (μAE) has received comprehensive attention since it can provide a much larger surface area and lead to higher response current [17], lower impedance [18] and limit of detection [19]. For the purpose of improving the performance of the μAE, previous researches have been carried out to investigate how the current response of the μAE are affected by the geometrical parameters, such as the micropillars shape [20], height [16], array density [17] and the relative angle between the micropillar and the flow direction [21]. However, these researches were only limited to improve the electrochemical performance of the μAE through optimizing one or several of these parameters, of which the influences on the current responses of the μAE didn’t get studied systematically.

In contrast with the conventional ED system, in which the mass transfer process of analytes involves only diffusion, detection through μEDS is usually performed under hydrodynamic conditions. Flowing sample solution brings consecutive analytes flux and a completely different electroactive species concentration distribution from that in static solution. More specifically, as the flow velocity increases, the diffusion layer near the electrode surface gets thinner, leading to a higher reactant concentration gradient and diffusion mass transfer rate, which finally result in an amplified electrode reaction current. For the convection-diffusion process in microchannel integrated with microelectrodes, distributions of the flow field and concentration field are in a coupled state and affected by both the flow rate and the geometrical parameters of microelectrodes [22]. Therefore, performance of the microelectrodes as well as the whole μEDS are usually evaluated with a consideration of both the geometrical and hydrodynamic conditions. Based on these two aspects, microelectrodes with the abovementioned conventional configurations have been extensively investigated. Most of the previous researches started with solving the governing equations, including Navier-Stokes equation describing the velocity field, convention-diffusion equation describing the concentration field and Butler–Volmer equation describing the electrode reactions, and aimed at identifying different mass transfer regimes [23,24,25], predicting a geometrical- or hydrodynamic-constrained limiting current response [26,27] and optimizing the configuration of microelectrodes [28,29].

Both analytical [30,31] and numerical [32,33,34,35,36] methods were used in previous studies. However, what most of these studies adopted were simplified 2D geometrical or mathematical models. For 2D μEDS such as the microband, of which the electrodes configuration is relatively simple, sufficiently accurate results are still achievable. But when the detector adopted in the μEDS is 3D μAE, through which a complex 3D flow runs, a serious of new characteristics such as the edge effect [37,38] and tail effect [39,40] arise and influence the mass transfer process in the microchannel. Therefore, to investigate the performance of the μAE under hydrodynamic conditions accurately, these details must be considered and adopting 3D numerical models to simulate the electrochemical behavior of the μAE is necessary.

The main objective of this study is to investigate the electrochemical behavior of the 3D micropillar array electrodes with different configurations under various hydrodynamic conditions in the microfluidic chip. To accomplish this purpose, a type of PDMS-based micropillar array electrode was fabricated and integrated into a microchip by using 3D printing and soft lithography technologies. Experiments of the cyclic voltammetry (CV) and chronoamperometry (CA) were performed and the results were used to validate the numerical modeling method. Based on numerical simulation, influences of the flow rate, array density, micropillar’s shape and size and the μAE’s layout on the current response were analyzed comprehensively. The tail effects in μAEs with different design parameters were quantitatively considered based on a pre-defined parameter of the current density ratio. This research should be instructive for the configuration and parameter design of microchip-based electrochemical detection systems.

## 2. Methods

### 2.1. Materials and Instrumentations

The polydimethylsiloxane (PDMS, SYLGARD™ 184) and its crosslinking catalyst were purchased from Dow Corning Corporation (Auburn, MI, USA). The UV-curable polymer for 3D printing was obtained from Young Optics, Inc. (Hsinchu, Taiwan). The Ag/AgCl ink for preparing the reference electrode was provided by BAS Inc. (Tokyo, Japan). The potassium ferricyanide (K_3_[Fe(CN)_6_]), potassium ferrocyanide (K_4_[Fe(CN)_6_]), potassium chloride (KCl) and 1H,1H,2H,2H-perfluorooctyltrichlorosilane were purchased from Macklin Biochemical Co., Ltd. (Shanghai, China). Ultrapure water (18.2 MΩ∙cm^−1^) was used for dilution and all experiments were carried out at room temperature. All chemicals were analytical grade and used without further purifications.

The positive masters of the microchannel and micropillars array were fabricated by the NanoArch P140 (BMF Precision, Shenzhen, China) and MiiCraft+ desktop DLP-SLA 3D printer (Young Optics, Inc., Hsinchu, Taiwan), respectively. The magnetron sputtering and oxygen plasma treatment were carried out using the PD-400 (Pudivaccum, Wuhan, China) and PDC-002 (Harrick, NY, USA), respectively. Electrochemical detections were performed through the CHI 760E electrochemical workstation (Shanghai Chen Hua Co., Ltd., Shanghai, China) with a three-electrode cell. A syringe pump (Lead Fluid Technology Co., Ltd., Baoding, China) was used to pump the analyte into the chip. The oven used in microchip fabrication process was provided by Taisite Instrument Co., Ltd. (Tianjin, China). A computer workstation (HP, Z8, G4, two Xeon Gold 6148 CPUs with 40 cores and 256 GB of RAM) was used for the numerical simulations.

### 2.2. Configuration of Microchip-Based Electrochemical Detection System (μEDS)

**Figure 1:**
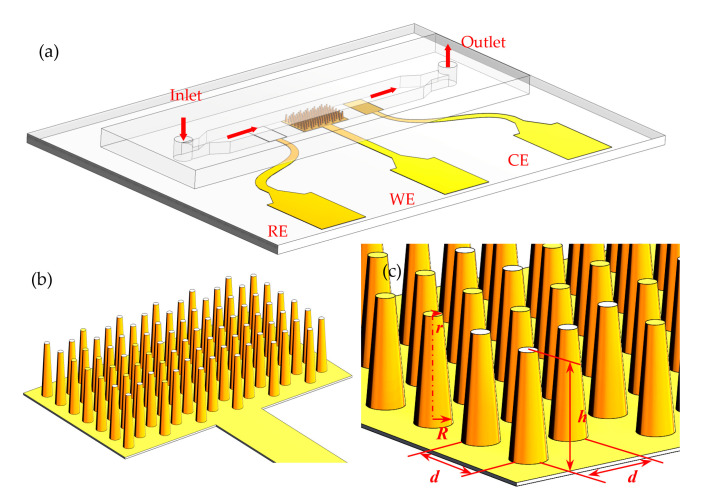
The schematic diagram of (**a**) the μEDS; (**b**) the μAE. (**c**) Blow-up view of the μAE with the definition of the geometrical parameters.

The μEDS, containing a working electrode (WE), a counter electrode (CE) and a reference electrode (RE), is as shown in Figure 1a. In this three-electrode system, a μAE was used as the WE; a Ag/AgCl ink-painted planar RE and a bare gold planar CE were placed at the two sides of the WE. All these three electrodes were integrated into a 350 μm-high microchannel. As demonstrated by Figure 1b,c, the μAE is composed of numerous micropillars constructed within a 1.5 × 2.5 mm^2^ planar area. The μAEs with varying micropillar heights (100, 200 and 300 μm) and spacing between two single micropillars (150, 200 and 250 μm) were modeled and investigated numerically, of which the specific parameters are listed in Table 1.

### 2.3. Numerical Simulation Method of the μEDS

#### 2.3.1. Theory

For the μEDS, a fast charge transfer is assumed for an electrochemical reaction in this study, and the mass transfer process of electroactive species in microchannel consists of two parts: the convection driven by velocity vector and the diffusion driven by the concentration gradient, as shown in Equation (1):

(1) $$\frac{\partial C}{\partial t}=D\nabla^{2}C-\vec{u}\cdot \nabla C$$

where *C* is the concentration of the analyte; *t* is the time; *D* is the diffusion coefficient; *u* is the flow velocity.

The redox reaction at the electrode surface is described as:

(2) $$O+ne^{-}⇌R$$

where *O* and *R* are the oxidized and reduced species, respectively. *n* is the number of transfer electron. Single-electron transfer occurs in this study.

The chronoamperometric current induced by the redox reaction can be predicted by the following Butler−Volmer equation [41]:

(3) $$i=nFA(k_{f}C_{O}(t)-k_{b}C_{R}(t))$$

where *F* is the Faraday’s constant; *A* is the area of the electrode; *C_o_*(*t*) and *C_R_*(*t*) are the concentration of the analyte at the electrode surface at time *t*. *k_f_* and *k_b_* are the forward and reverse reaction rate constants, which can be expressed as:

(4) $$k_{f}=k_{0}\exp\left(-\alpha \frac{F}{RT}(E-E^{0’})\right)$$

(5) $$k_{b}=k_{0}\exp\left(\left(1-\alpha )\frac{F}{RT}(E-E^{0\prime }\right)\right)$$

where *k*_0_ is the standard heterogeneous rate constant; *α* is the transfer coefficient; *R* is the gas constant; *T* is the absolute temperature; *E* is the potential applied to the electrode; *E*^0′^ is the equilibrium potential.

#### 2.3.2. Numerical Model

**Figure 2:**
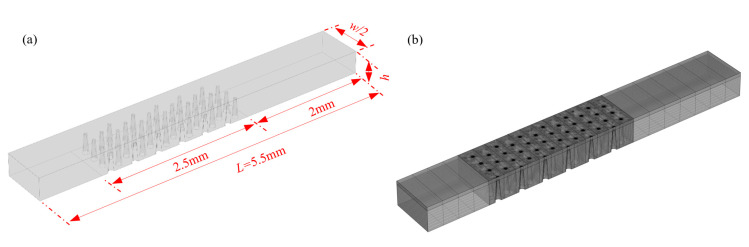
(**a**) The computational domain of the μEDS; (**b**) The schematic diagram of the meshing method.

As the microchannel integrated with the μAE is actually symmetric, computational domains corresponding with half of the fluid region were adopted, which were then discretized based on a structural hexahedral mesh. The schematic diagrams of the computational domain and grid are as shown in Figure 2a,b, respectively. By means of this method, the numerical model of the μAEs of which the micropillars are different in height, spacing, shape and arrangement form (Table 1), were built and then solved to obtain the steady-state current response. The inlet boundary with a constant reactant concentration of 5 mM, the outlet boundary of atmospheric pressure and the flux-free condition of the other boundaries except the electrode surface were adopted in all calculating examples, which covers a flow rate range of 0 to 30 μL/min. All parameters for the numerical simulation were listed in Table 2. Both the modeling and solving process were accomplished through COMSOL Multiphysics 5.4 (COMSOL Inc., Stockholm, Sweden).

### 2.4. Fabrication of μEDS

**Figure 3:**
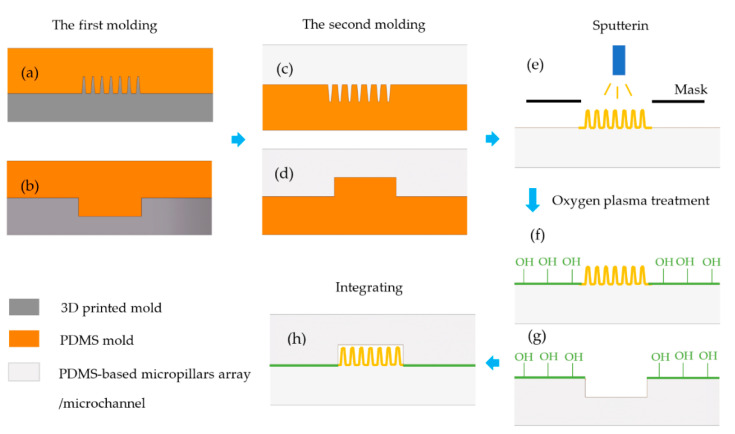
The fabrication process of the μEDS. (**a**,**b**): fabrication of the negative PDMS masters of μAE and microchannel; (**c**,**d**): fabrication of the μAE and microchannel; (**e**): deposition of the conducting layer; (**f**,**g**): oxygen plasma treatment; (**h**): integration of the detection microchip.

**Figure 4:**
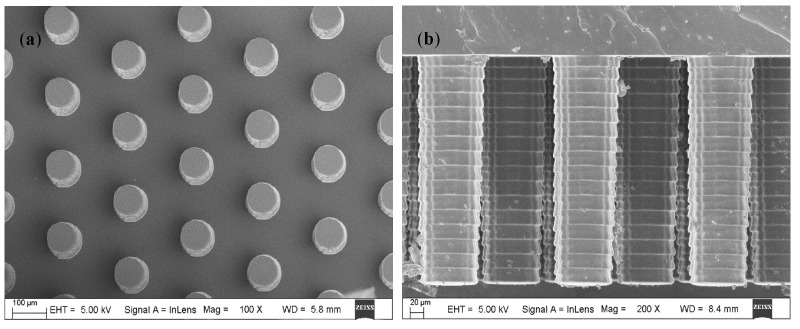
The SEM images of fabricated micropillars. (**a**) top view of micropillars; (**b**) side view of micropillars.

The fabrication process of the μEDS is illustrated in Figure 3. UV-curable polymer positive masters of the micropillar array (Figure 4) and microchannel were fabricated through two different high-precision 3D printers, as mentioned previously. The PDMS-based micropillar arrays were then manufactured by soft lithography [42,43]. Next, the 3D-printed masters were put into a vacuum desiccator, into which a few drops of 1H,1H,2H,2H-perfluorooctyltrichlorosilane were added, and vacuumed for 30 min to form a silane compound layer on the maters’ surface. Sylgard 184 PDMS polymer was mixed fully with its crosslinking catalyst at 10:1 (weight: weight) and degassed by vacuum for 45 min, and the mixture was cast against the 3D printed molds and polymerized at 60 °C for more than 2 h (Figure 3a,b). After curing, the negative PDMS replicas were peeled off and employed as masters to mass-produce the PDMS micropillar arrays and microchannels (Figure 3c,d).

Magnetron sputtering procedure was then introduced to get the μAEs. Specifically, the PDMS micropillar arrays were covered by steel masks with a hollowed-out shape of the electrode (as shown in Figure 1) and then placed in a sputter. The chromium adhesion layer was firstly deposited with the power of 100 W, duration time of 200 s, and then the gold was subsequently deposited with the power of 300 W, duration time of 500 s to form the conducting layer eventually (Figure 3e). The flow rate of the argon gas in sputter was always 50 sccm.

After the deposition process, one of the two planar electrodes, which works as the solid-state reference electrode, was printed by the Ag/AgCl ink and then baked in the oven at 120 °C for 5 min. The micropillars array and microchannel were then placed in an oxygen plasma for 30 s (45 W at 0.46 Torr). Then these two oxygen plasma-treated parts were joined together quickly and baked at 60 °C for more than 2 h to form an irreversible bonding.

For the fabrication of the micropillar array, the conventional method is based on the photolithography [17,18,44], which shows better definition and reproducibility. However, the micropillar height is limited by the lithography process [16], which makes it difficult to acquire micropillars with a high aspect-ratio to increase the detection sensitivity. Moreover, compared with the 3D printing and soft lithography, the photolithography process is relatively complex and expensive. Hence, the fabrication method in this study is a more effective way to acquire low-cost μAE.

### 2.5. Experiments of the Electrochemical Detection

Through the above mentioned process, the contrastive planar microelectrodes and the μAEs (μAE200) in which the cylindrical micropillars are 300 µm high and the spacing between each adjacent two of them are 200 µm, were manufactured and their electrochemical performance was investigated based on the cyclic voltammetry (CV) and chronoamperometry (CA). The schematic diagram of the electrochemical detection system is shown in Supplementary Materials Figure S1.

A solution of 5 mM K_3_[Fe(CN)_6_]/K_4_[Fe(CN)_6_] with 0.1 M KCl were used in all experiments of this research. The CV experiments were firstly performed with the flow rate of zero, the scan rate of 0.05 V/s and the voltage range of −0.2 to 0.6 V. Then the potential corresponding with the peak current of the cyclic voltammogram was applied to the working electrode to perform the CA experiments, in which the flow rates in the microchannel varied from 0 to 30 μL/min. Finally, the steady-state response current of the CA experiments was recorded for the further analysis.

## 3. Results and Discussion

### 3.1. Effect of Flow Rate and Spacing

Micropillars of the μAE are all located in a specified area (1.5 × 2.5 mm^2^), therefore the number of micropillars and the total surface area of μAE are confined by the spacing between two adjacent micropillars. The effect of the spacing on the current response was analyzed. Besides the height of micropillar, which was set as a constant 300 μm, the other parameters of μAEs used for numerical study were the same as listed in Table 1, and as a reference, the planar electrode was also considered.

**Figure 5:**
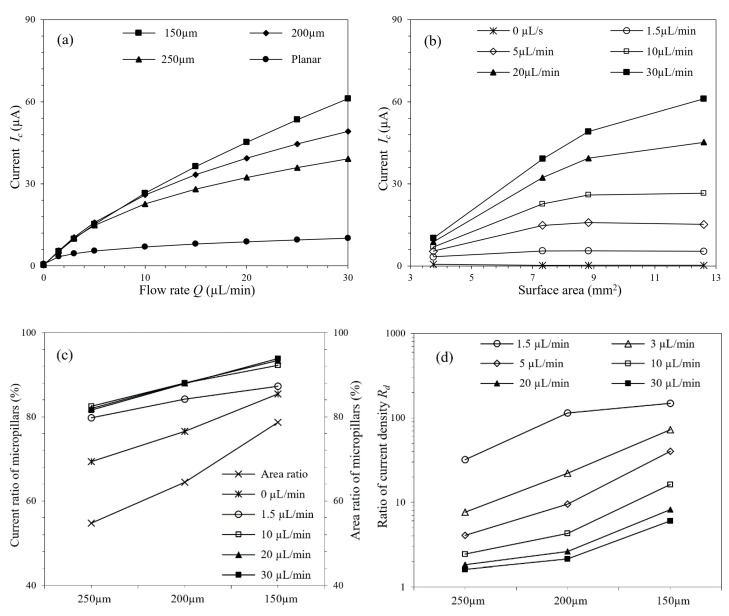
(**a**) Current responses of μAEs with various spacings at different flow rates and spacings; (**b**) Current responses of μAEs with various surface area at different flow rates; (**c**) Current and area ratios of μAEs with various spacings at different flow rates; (**d**) Ratios of the current density between the first and last row of micropillars.

Figure 5a,b show the current responses at different flow rates and spacings (surface areas). As demonstrated by the figures, increased flow rate leads to a higher response current, and for μAEs, this increment is more significant. For the stationary condition, the response current of μAE increases with the spacing between the micropillars, and the planar electrode yields a higher current response than μAE. But when the flow rate is 30 µL/min, the response current of μAE with the spacing of 150 µm gets 6.03 times larger compared with the planar electrode. The concentration distribution of μAE at different flow rates is shown in Figure S2. It can be seen that the range of low concentration becomes smaller when the flow rate increases. Higher flow rate leads to decreased thickness of the diffusion layer, increased concentration gradient and mass transfer rate on the electrode’s surface, and results in more significant response current, eventually.

The surface of μAE can be divided into two parts: the planar base surface and the micropillars surface. Both these two parts contribute to the total response current. In this study, the area ratio is defined by the proportion of micropillars surface area in the total surface area of μAE. Similarly, the current ratio is defined by the proportion of micropillars response current to the total response current of μAE. Figure 5c presents the current ratio and area ratio of μAE with different spacings. With the increase of flow rate, the current ratios increase firstly and then tend to be steady (>10 µL/min). The results show that the most part of the response current derives from the micropillars surface, which contributes more than 80% of the total current response at the flow rate of 1.5 µL/min. Even under the condition of zero flow rate, this ratio is still more than 60%. And in addition, as reflected by Figure 5c, for each μAE mentioned the current ratio is always larger than the area ratio, which further indicates the major role of the micropillars surface in generating the current response.

For the μAE, another characteristic existing under hydrodynamic conditions is the tail effect, namely that the electrochemical performance of the downstream micropillars are affected by the upstream counterparts [40]. To quantitatively analyze the tail effect, ratios of current density between the first and last row of micropillars (R_d_) were defined and calculated, as shown in Figure 5d. Obvious tail effect, which is characterized by the large current density ratio, is usually existed in cases where the spacings of μAEs are small (150 or 200 µm for instance) or the flow rates are relatively low (<10 µL/min). In such circumstances, as shown in Figures S2a and S3a, there is a wide range of low concentration downstream, and the concentrations detected by the front and back micropillars, as well as the corresponding current responses, differ greatly. This feature restricts the benefits of the μAE brought by the increased reaction area, and further increment of the micropillars number doesn’t lead to improved detection performance.

### 3.2. Effect of Micropillar Height

**Figure 6:**
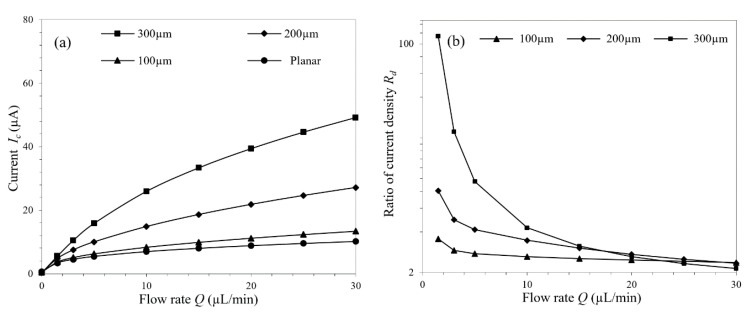
(**a**) Current responses of the planar electrode and the μAEs with micropillars of different heights under varying flow rates; (**b**) The current density ratios of the μAEs with micropillars of different heights under varying flow rates.

Besides the spacing between micropillars, another factor affecting the μAE’s surface area is its height. The variation trend of the μAEs’ current responses along with the micropillars height is shown in Figure 6a. Increasing the micropillars height leads to higher responses, and this trend is more obvious when the micropillars are already relatively high. Or in other words, compared with the planar electrode, advantage of the μAE doesn’t appear when the micropillars are low. For example, there is little difference between the current responses of the μAE with micropillars 100 µm in height and the planar electrode.

Based on the pre-defined current density ratio, the tail effect in μAEs with the micropillars of varying heights were investigated. When the flow rate is relatively high, the tail effect weakens, and each μAE exhibits nearly the same current density ratio regardless of the micropillars height, as shown in Figure 6b. Hence, differences of the tail effects in μAEs with varying micropillars height are mainly reflected in cases of low flow rates (5 µL/min, for instance), where the μAE with higher micropillars demonstrates larger current density ratio. Increased micropillars height brings larger surface area and more analytes participating the electrode reaction, and eventually leads to higher current response. Figure S4 shows the concentration distributions in μAE with micropillars of different heights at the flow of 5 µL/min. When the micropillar is relatively high, 300 µm for instance, most of the influent analytes were consumed by the upstream micropillars and downstream low concentration spreads widely.

### 3.3. Effect of Micropillar Layout

Two types of μAE layout were investigated in this work. In the same base area, identical numbers of micropillars were distributed in staggered and aligned arrangements. The mechanism of the mass transfer intensification by flow has been analyzed previously, but the concrete effects of the flow rate on μAEs with these two different layouts are different. In the cases with low flow rates (<15 µL/min), where the overall downstream concentration is already relatively low, the fluid concentration around the last row of the staggered micropillars is even lower than that of the aligned micropillars. As the flow rate increases, all of the micropillars begins to get higher mass transfer flux due to the higher ambient concentration gradient, and the current responses are also improved consequently. For the μAE with staggered micropillars, this trend has been quantitively analyzed in Section 3.1. But for the μAE in aligned layout, benefits of the flow are impaired, which is embodied in that the degrees to which the downstream micropillars current response increases are smaller compared with the upstream micropillars. When the micropillars are aligned, more analytes pass through any two adjacent columns of micropillars and can hardly participate in the reaction (Figure S5). Therefore, when the flow rate is relatively high (>15 µL/min), the current response of the last row of the aligned micropillars is lower than that of the staggered micropillars.

**Figure 7:**
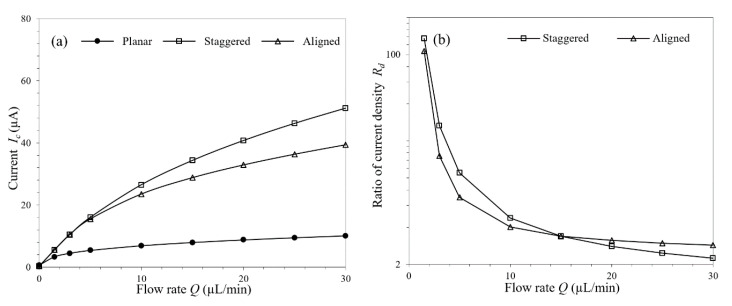
(**a**) Current responses of the planar electrode and the μAEs in different layouts under varying flow rates; (**b**) The current density ratios of the μAEs in different layouts under varying flow rates.

These two similar but not identical variation trends had been summarized and demonstrated in Figure 7b, where an intersection of the current density-flow rate curves appears at the flow rate of approximately 15 µL/min. It’s worth mentioning that this slight difference is precisely due to the fact that the staggered micropillars are more advantageous in getting the electroactive species involved in the electrode reaction. Therefore, it can be concluded that the aligned layout is not an ideal option because of the defective current response, as shown in Figure 7a.

### 3.4. Effect of Micropillar Shape

Different shapes of the micropillars including cone, triangle, square, and cylinder were studied in this work, and the cross-sections of the micropillars in these shapes are shown in Figure S6 and the corresponding geometrical parameters are listed in Table S1.

**Figure 8:**
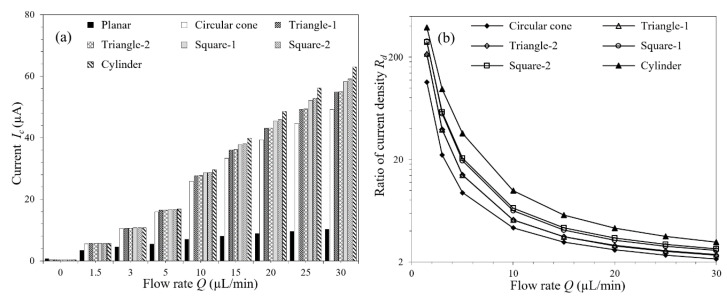
(**a**) Current responses of the planar electrode and the μAEs with micropillars in different shapes under varying flow rates; (**b**) The current density ration of the μAEs with micropillars in different shapes under varying flow rates.

The response currents of the μAEs with micropillars in different shapes are shown in Figure 8a. It shows that the μAE, whatever the micropillars shape, has a significant advantage in the current response under hydrodynamic conditions compared with the planar electrode, and this advantage becomes more obvious with the increased flow rates. For example, the μAEs have a more than 4.85-fold response current compared with the planar electrode, at the flow rate of 30 µL/min.

Although there is almost no difference between the current responses of the μAEs with the micropillars in different shapes at low flow rates (5 µL/min, for instance), the μAE with cylindrical micropillars shows better performance than μAEs with micropillars in the others shape as the flow rates increase to a relatively high value (30 µL/min, for instance). Moreover, different relative angles between the micropillars and the flow direction were also considered, which were proven to have almost no influence on the current responses. As shown in Figure 8a, the μAEs with the micropillars of Triangle-1 and Triangle-2 yields almost the same current responses at all flow rates investigated, and the current responses of the μAEs with the Square-1 and Square-2 micropillars differ by only approximately 1.3%, even at the high flow rate of 30 µL/min.

As for the tail effect, it was found that the variation trend of the degree along the flow rates to which the tail affects the current responses are uniform in the μAEs in the default staggered layout, whatever the micropillars shape. This conclusion can be represented by the monotonically decreasing current density ratio as the flow rates increases, as shown in Figure 8b. Different from Figure 7b, there is no crossing between the current density ratio-flow rate lines in Figure 8b, which indicates that changing the micropillars shape leads to a uniform shift of all the micropillars current responses but the relative magnitudes among them remain the same.

### 3.5. Experimental Verification

**Figure 9:**
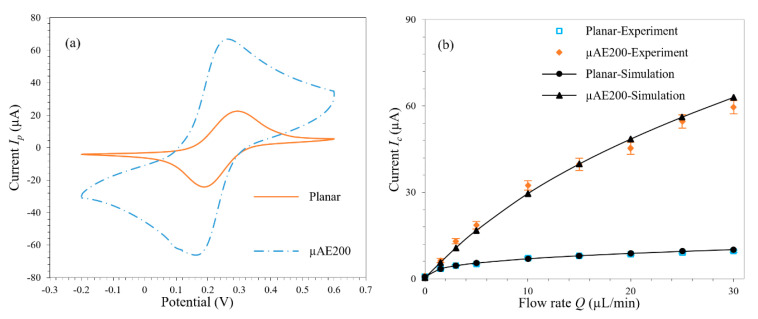
(**a**) Experimental CV of the planar microelectrode and μAE200 at the scan rate of 0.05 V/s; (**b**) Experimental and simulated current response of the planar microelectrode and μAE200 at different flow rates; In the 5 mM K_3_[Fe(CN)_6_]/K_4_[Fe(CN)_6_] solutions with 0.1 M KCl vs. Ag/AgCl.

Results of the CV experiments are shown in Figure 9a. The μAE200 demonstrates a much larger peak current compared with the planar microelectrode, which proves the effectiveness of the μAEs. Based on the CV results, the 0.25 V corresponding with the peak current was adopted as the working electrode potential to conduct the CA experiments, and the steady-state currents of both the planar microelectrode and μAE200 at different flow rates (0 to 30 μL/min) were recorded, as shown in Figure S7. A good agreement was found between the experimental data and the numerical simulation results of the response currents, which proved the validity and the accuracy of the numerical method, as shown in Figure 9b.

## 4. Conclusions

The performance of μAEs with micropillars of different spacing, height, shape, and arrangement form was investigated under varying hydrodynamic conditions based on numerical simulation. It was found that the μAE, regardless of the specific shape of the micropillar, presents a much higher current response under flow conditions compared with the planar microelectrode and this advantage gets more pronounced as the flow rate increases. The μAE with cylindrical micropillars shows better performance than μAEs with micropillars in the other shapes at a relatively high flow rate and the relative angles between the micropillars and the flow direction have little impact on the current responses. Higher micropillars in the μAE bring more significant current response compared with the planar microelectrode due to the increased reaction area and this advantage is only apparent when the micropillars are relatively high. In respect of the current response, the μAE in staggered layout performs better the μAE in aligned design at any flow rate. The tail effect limits the current responses of the downstream micropillars as well as the whole μAE, and this negative consequence diminishes as the flow rate increases. The experimental data are highly consistent with the numerical simulation results, which proves the accuracy and the effectiveness of the numerical simulation method.

This work provides an applicable guideline for parameters design and structure optimization of the μEDS. In addition, nanochip-based electrochemical detection system (nEDS) with nanoelectrode in nanoflows is one of main research directions recently [45,46], which has the characteristic [47,48,49,50] of lower reagent consumption, faster analysis time and larger surface-to-volume ratio comparted with μEDS. The presented method in the study can be applied to analyze and optimize the electrochemical performance of nEDS.

## Figures and Tables

**Table 1:** Parameters of the working electrode for numerical study.

Parameters	Planar	Conical Micropillar		
Projection area *l* × *w* (mm^2^)	1.5 × 2.5			
Top radius *r_t_* (μm)	-	25		
Base radius (μm)	-	50		
Height *h* (μm)	-	100/200/300		
Spacing *d* (μm) ^1^	-	150	200	250
Number of pillars *n*	-	136	78	55
Surface area *S* (mm^2^)	3.75	7.33	8.82	12.60
Area ratio ^2^ *S*_g_	1.0	1.95	2.35	3.36

^1^ Spacing between the centers of two adjacent micropillars. ^2^ The ratio of the active area between the μAE and the planar electrode.

**Table 2:** Parameters for the numerical simulation.

Parameters	Unit	Value
Diffusion coefficient *D*	m^2^/s	6.5 × 10^−5^
Faraday’s constant *F*	*C*/mol	96,485.33
Standard heterogeneous rate constant *k*_0_	m/s	1 × 10^−4^
Transfer coefficient α	-	0.6
Gas constant *R*	J/(mol·K)	8.314
Absolute temperature *T*	K	298.15
Applied potential *E*	V	0.25

## Supplementary Materials

The following are available online at https://www.mdpi.com/2072-666X/11/9/858/s1, Figure S1: The schematic diagram of the electrochemical detection system, Figure S2: Concentration distribution in μAE with the spacing of 200 µm at different flow rates, Figure S3: Concentration distribution in μAE with different spacings at the flow rate of 10 µL/min, Figure S4: Concentration distribution of the μAEs with micropillars of different heights and the planar electrode at the flow rate of 5 µL/min, Figure S5: Concentration distribution of the μAEs in different layouts at the flow rate of 10 µL/min, Figure S6: Cross-section of the micropillars in different shapes, Figure S7: Experimental CA of the planar electrode and μAE200 with different flow rates, Table S1: Parameters of the working electrode with different shapes.
