# Supplementary material for: Electrochemical Performance of Micropillar Array Electrodes in Microflows
Source: Micromachines (Basel). 2020 Sep 17;11(9):858. doi: 10.3390/mi11090858 (PMC7570346; doi:10.3390/mi11090858)
Supplement: Supplementary file 1 [file micromachines-11-00858-s001.pdf]

Supplementary Material

Electrochemical Performance of Micropillar Array Electrodes in Microflows

Bo Liu, Chuanwen Lv, Chaozhan Chen, Bin Ran, Minbo Lan, Huaying Chen and Yonggang Zhu

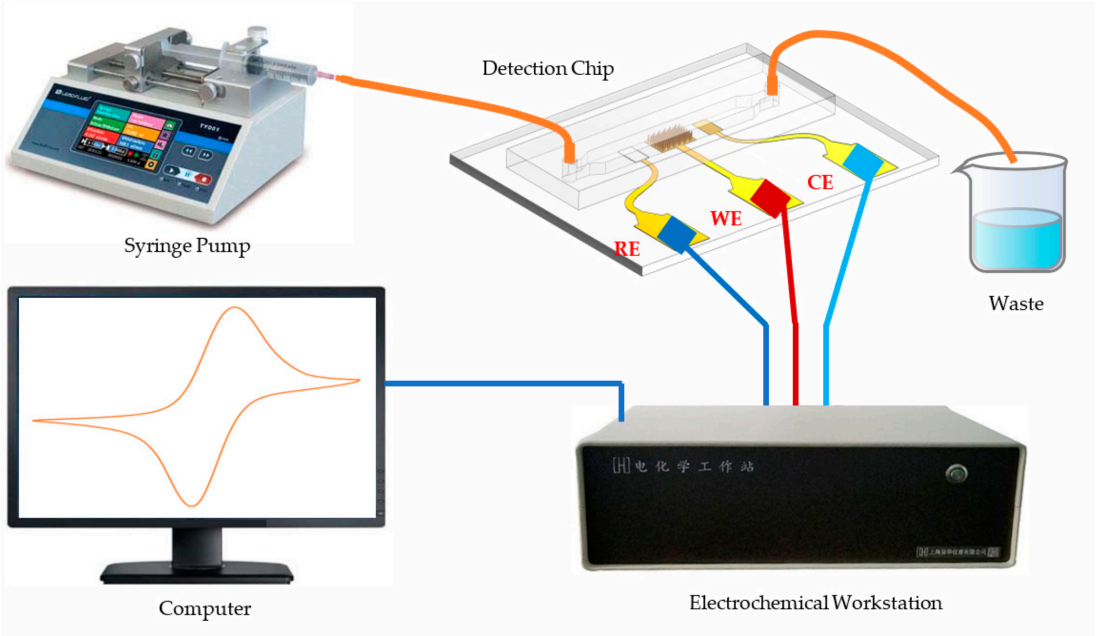

Figure S1. The schematic diagram of the electrochemical detection system.

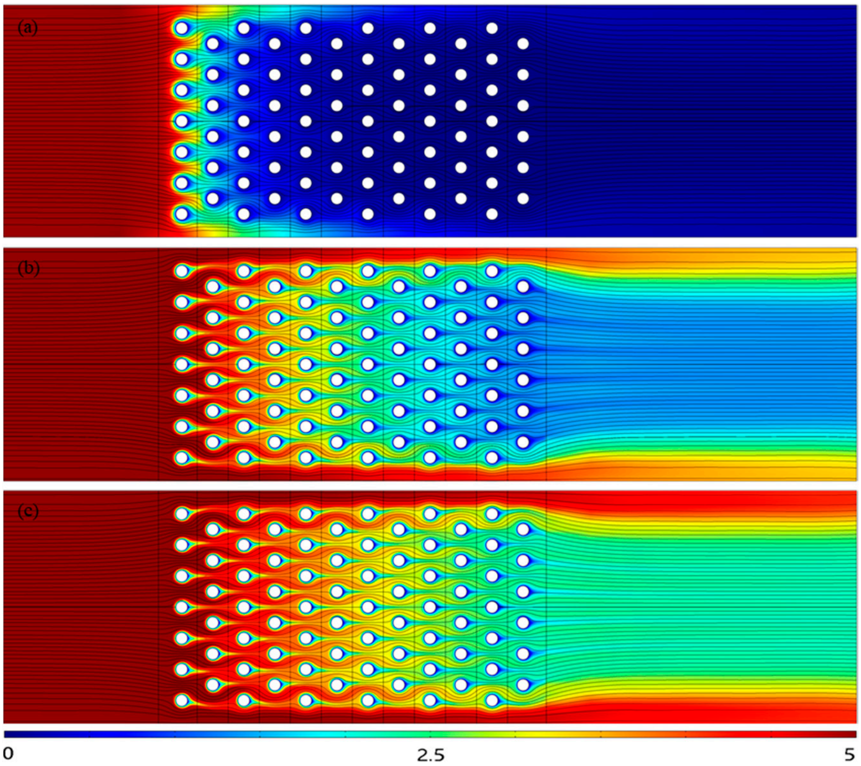

Figure S2. Concentration distribution in μAE with the spacing of 200 μm at different flow rates: (a) Q=1.5 μL/min; (b) Q=15 μL/min; (c) Q=30 μL/min.

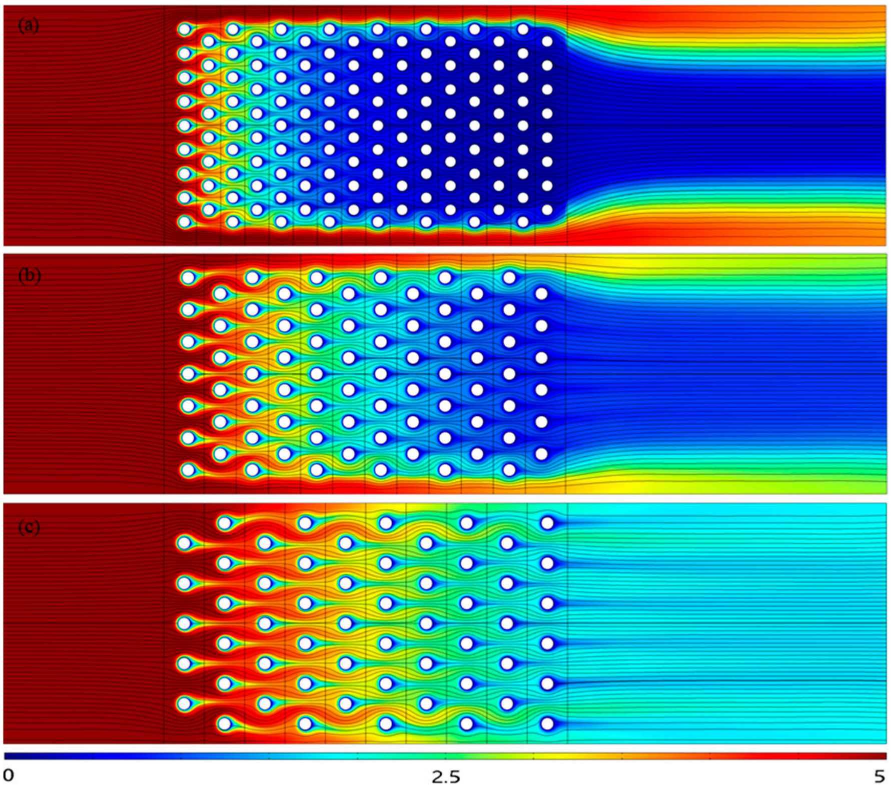

Figure S3. Concentration distribution in μAE with different spacings at the flow rate of 10 μL/min: (a) d=150 μm; (b) d=200 μm; (c) d=250 μm.

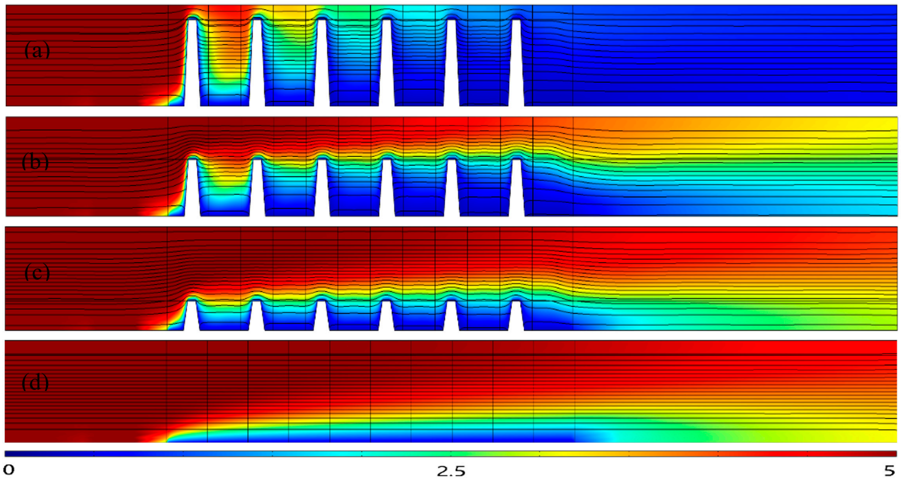

Figure S4. Concentration distribution of the μAEs with micropillars of different heights and the planar electrode at the flow rate of 5 μL/min: (a) h=300 μm; (b) h=200 μm; (c) h=100 μm; (d) planar.

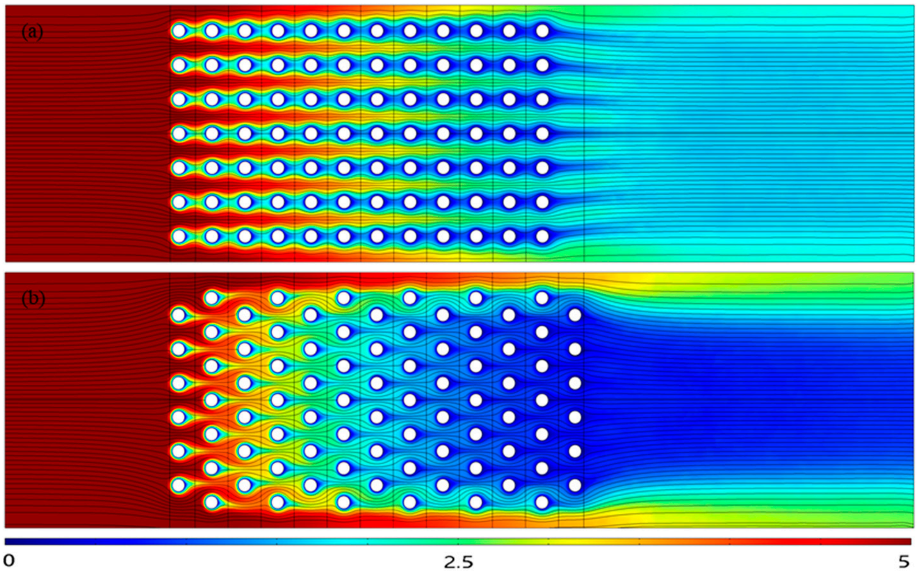

Figure S5. Concentration distribution of the μAEs in different layouts at the flow rate of 10 μL/min: (a) Aligned; (b) Staggered.

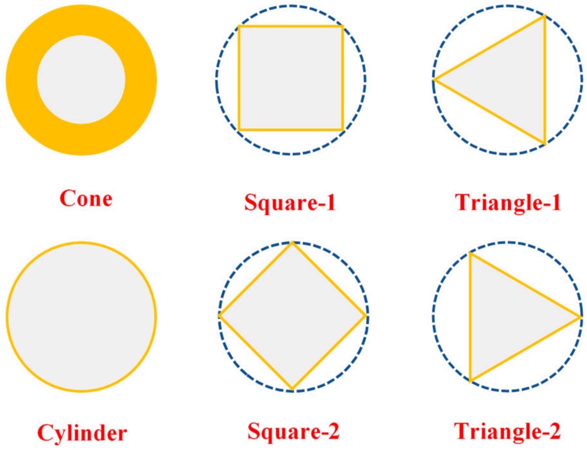

Figure S6. Cross-section of the micropillars in different shapes with the same base radius (50μm).

Table S1. Parameters of the working electrode with different shapes.

Parameters	Planar	Cone	Cylinder	Square	Triangle
Projection area $l \times w$ (mm^2)			1.5×2.5		
Height h (μm)	-		300		
Spacing d (μm) [1]	-		200		
Number of pillars n	-		78		
Top radius r_t (μm)	-	25	50	-	-
Base radius (μm)	-	50	50	-	-
Side length (μm)	-	-	-	70.71	86.60
Surface area S (mm^2)	3.75	8.82	11.10	10.37	9.83
Area ratio [2] S_g	1.0	2.35	2.96	2.77	2.62

[1] Spacing between the centers of two micropillars.

[2] The ratio of the active area between the μAE and the planar electrode.

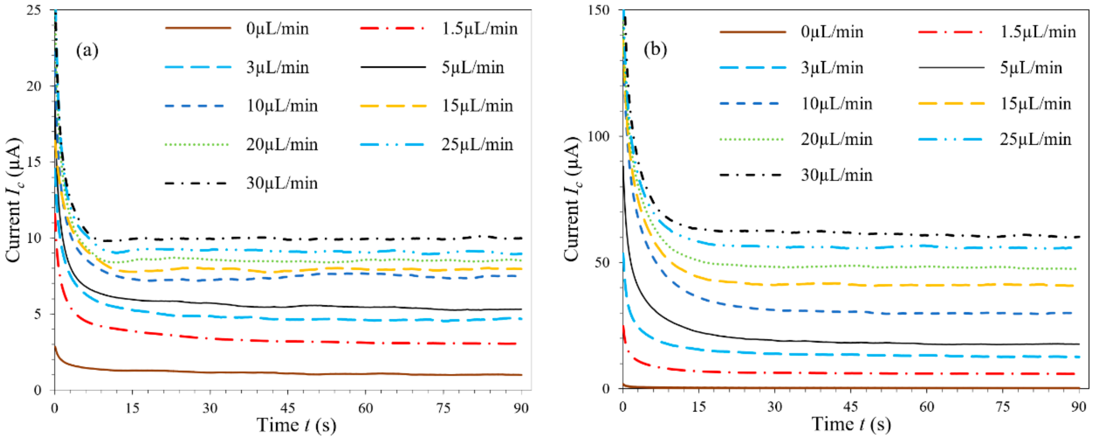

Figure S7. Experimental CA of (a) the planar electrode and (b) μAE200 at different flow rates; In the 5 mM $K_3[Fe(CN)_6]/K_4[Fe(CN)_6]$ solutions with 0.1 M KCl vs Ag/AgCl.

© 2020 by the authors. Submitted for possible open access publication under the terms and conditions of the Creative Commons Attribution (CC BY) license (http://creativecommons.org/licenses/by/4.0/).
